# Establishing a Metabolite Extraction Method to Study the Metabolome of *Blastocystis* Using NMR

**DOI:** 10.3390/molecules26113285

**Published:** 2021-05-29

**Authors:** Jamie M. Newton, Emma L. Betts, Lyto Yiangou, Jose Ortega Roldan, Anastasios D. Tsaousis, Gary S. Thompson

**Affiliations:** 1 Laboratory of Molecular & Evolutionary Parasitology, RAPID Group, School of Biosciences, University of Kent, Canterbury CT2 7NJ, UK; jmn24@kent.ac.uk (J.M.N.); elb48@kent.ac.uk (E.L.B.); lytoyiangou@hotmail.com (L.Y.); 2 Wellcome Trust Biomolecular NMR Facility, School of Biosciences, University of Kent, Canterbury CT2 7NJ, UK; J.L.Ortega-Roldan@kent.ac.uk

**Keywords:** *Blastocystis*, ^1^H NMR, metabolite extraction, metabolomics

## Abstract

*Blastocystis* is an opportunistic parasite commonly found in the intestines of humans and other animals. Despite its high prevalence, knowledge regarding *Blastocystis* biology within and outside the host is limited. Analysis of the metabolites produced by this anaerobe could provide insights that can help map its metabolism and determine its role in both health and disease. Due to its controversial pathogenicity, these metabolites could define its deterministic role in microbiome’s “health” and/or subsequently resolve *Blastocystis*’ potential impact in gastrointestinal health. A common method for elucidating the presence of these metabolites is through ^1^H nuclear magnetic resonance (NMR). However, there are currently no described benchmarked methods available to extract metabolites from *Blastocystis* for ^1^H NMR analysis. Herein, several extraction solvents, lysis methods and incubation temperatures were compared for their usefulness as an extraction protocol for this protozoan. Following extraction, the samples were freeze-dried, re-solubilized and analysed with ^1^H NMR. The results demonstrate that carrying out the procedure at room temperature using methanol as an extraction solvent and bead bashing as a lysis technique provides a consistent, reproducible and efficient method to extract metabolites from *Blastocystis* for NMR.

## 1. Introduction

*Blastocystis* is a genus of anaerobic protozoan that resides in the gastrointestinal tract of many vertebrate species and has historically been classified as a parasite, yet its pathogenicity has been a subject of dispute in recent years. *Blastocystis* has a unique metabolism and possesses a mitochondrial-related organelle (MRO) with chimeric characteristics of an aerobic mitochondrion and hydrogenosomes [1]. Many of these characteristics have been acquired by lateral gene transfer from prokaryotes and possibly other eukaryotic organisms in the gastrointestinal tract, and these have likely supported the adaptation of *Blastocystis* to the gut environment [2].

Previous in vitro studies aimed at mapping the unique metabolic pathways in *Blastocystis* have been based on genome and transcriptome analyses [3,4,5]. Biochemical analysis has involved fractionation, the separation of organelles by isopycnic density and the analysis of absorbance following the addition of certain substrates [6]. The latter of these approaches monitors enzyme activity in different organelles based on available nutrients and added substrates in vitro. This approach is limited in the range of enzymes and pathways that can be monitored. Therefore, a technique in which the whole metabolome can be analysed in the context of the host or in vitro culture is required. Metabolomics is a technique which can be utilised to analyse the metabolome of a cell or microorganism. This technique has been used to analyse the metabolomes of many microbes [7,8], plants [9], nematodes [10] and animal cells [11,12,13]. Additionally, it has also been used to detect the molecules present in biological liquids such as blood [14], urine [14,15,16] and breast milk [17]. Mass spectrometry (MS) is probably the most popular analysis method for the detection and characterisation of small molecules and has been extremely successful because of its high sensitivity [10,18]. However, its arduous sample preparation can involve many steps to produce samples with good ionisation and MS properties. Subsequently, this can result in a loss of sample and the integrity of the metabolites being prejudiced. Therefore, reproducibility and accurate quantification can be difficult to achieve. In contrast, NMR can provide a simpler, more reproducible method for quantitative molecule detection, albeit with considerably lower sensitivity. NMR does not require the same laborious sample preparation that MS does, and the sample can remain intact throughout the analysis, thus making it a better quantitative tool [19,20,21,22,23,24]. However, for reasons of practicality and health and safety, NMR methods still require the extraction of metabolites from semi-solid samples such as cell cultures, as high resolution ^1^H NMR is a solution state method. The question then becomes which solvent and method should be used to best isolate the desired group of molecules from a sample. For example, methanol is commonly used to extract polar molecules [10,11,13], while chloroform is commonly used to extract non-polar molecules [10,11].

Currently, the only protozoan parasite to have its metabolome analysed by NMR is *Giardia lamblia* [7]. In this study, the metabolome of *G. lamblia* was analysed by high resolution ^1^H magic angle spinning (HR-MAS) NMR. HR-MAS does not require an extraction solvent as the cells remain intact [7]. However, HR-MAS experiments have some major drawbacks: firstly, they require a relaxation filter to exclude larger molecules such as proteins, as these produce a background unfavourable for the integration of sharper peaks, thus hampering quantification and comparison. [25]. The presence of this relaxation filter affects the sensitivity of the experiment and reduces the number of metabolites that can be detected. Secondly, HR-MAS experiments are limited by the volumes and quantities of samples that can be run with a maximum of 50 µL, which is at least ten times lower than the volumes usually used in liquid state NMR.

^1^H NMR spectra have a proven track record for metabolite analysis from a number of biofluids and extraction methods [10,11,12,13,14,15,17]. Therefore, a combination of ^1^H NMR metabolomics using a 1D-^1^H-NOESY pulse sequence with an extraction protocol that only extracts small molecules provides an effective method for mapping *Blastocystis* metabolic pathways.

Herein, we aimed to investigate different extraction approaches in order to develop the optimum step-by-step method to extract metabolites from *Blastocystis* for analysis via ^1^H NMR in order to analyse its metabolism.

## 2. Materials and Methods

### 2.1. Blastocystis Culture

*Blastocystis* ST7 cultures were grown axenically in 8 mL of Iscove’s Modified Dulbecco’s Medium (IMDM) (Gibco-Catalogue no 12,200,069 Thermo Fisher scientific) with 10% heat-inactivated horse serum (HIHS) (Gibco-Catalogue no 26,050,088 Thermo Fisher scientific). All cultures were passaged every 3–4 days depending on their growth rate and were subsequently expanded. All cultures were incubated at 37 °C in 95% CO_2_ and 5% O_2_. The gas concentration was maintained by a gas pack (BD-Catalogue no 261205) in an anaerobic chamber (Oxoid-Product code 10,107,992 Fisher scientific). Cell counts were achieved manually using a Neubauer haemocytometer (Brand-Catalogue no 717810).

### 2.2. Cell lysis and Metabolite Extraction

*Blastocystis* cultures intended for metabolite extraction were pooled in a 50 mL tube and centrifuged at 1000× *g* for 5 min at 4 °C and the supernatant was discarded. Resulting pellets were re-suspended in 5 mL of Locke’s solution and given 2× washes with Stone’s modification of Locke’s solution (ATCC medium 1671), which was removed by a subsequent centrifugation at 1000× *g* for 5 min at 4 °C. The washed pellets were snap frozen in liquid nitrogen and stored at −80 °C.

Three steps were implemented for each experiment to determine the optimum extraction protocol and were each repeated four times. The conditions of each of the 4 experiments are shown in Table 1.

Step 1: Three cell cultures were thawed, resuspended in 5 mL of Lockes’ solution and then homogenised by vortexing for 30 s. These were then divided into two equal weight batches for parallel analysis. Each batch was centrifuged at 1000× *g* for 5 min at 4 °C, after which the supernatant was removed.

Step 2: The two batches were added to one of two different solvents: either 4 mL of ethanol:water (3:1) or 4 mL of methanol:water (1:1). The two different solvent batches were further processed at either −20 °C, room temperature (RT) or 60 °C (with samples for each solvent at each of the three temperatures). Each batch was then disrupted using one of two methods; either sonication in 3 × 30 s bursts or bead bashing by vortexing with 200 mg of 0.4 mm glass beads for 30 s followed by a 3-min incubation at either −20 °C, RT or 60 °C, then followed by vortexing for a further 30 s. 

Step 3: Resulting solutions were then divided into 4 × 1 mL aliquots and centrifuged for 15 min at 4 °C at 10,000× *g*. The supernatants were decanted into fresh tubes and lyophilised.

### 2.3. Preparation for ^1^H NMR Acquisition

The lyophilised desiccates were suspended in 330 μL of milliQ H_2_O, then vortexed for 30 s. The four supernatants of each sample where recombined and 147 μL of D_2_O containing 5 mM of non-deuterated DSS was added, resulting in a final DSS concentration of 0.5 mM.

### 2.4. Analysis of Aqueous Extracts by ^1^H NMR Spectroscopy

One-dimensional (1D) ^1^H spectra were obtained using a 600 MHz Avance III NMR spectrometer (Bruker) with a QCI-P cryoprobe with experiments measured at an calibrated temperature of 298K. Temperatures were calibrated using the residual protonated peaks from MeOH in a D4-MeOH sample to avoid radiation damping effects from the high Q value of the QCI-P cryoprobe used [26,27]. For each sample, the spectrometer was locked to D_2_O and the experiments were measured automatically using ICON NMR and a set of custom macros. Calibrations were carried out for each sample using a short excitation sculpting experiment; these included automated tuning and matching, measurement of the water offset and 90° pulse calibration, which was made using the stroboscopic nutation method of Wu and Otting [28]. The soft pulse power levels were calculated based on attenuated values calculated from the 90° pulse. The receiver gain measured for each sample and was limited to a maximum value of 128. A 1D-^1^H NOESY 100 ms mixing time was run. Data were accumulated over 512 scans with eight dummy scans. A spectral width of 12.02 ppm (7211 Hz) was used, and 32,768 data points were acquired, giving an acquisition time of 2.27 s. Acquisitions were separated by a relaxation delay of 3 s. The relaxation delay was increased, and the acquisition time decreased to provide sufficient water suppression.

### 2.5. Processing and Analysis of ^1^H NMR Data

All NMR spectra were phased, manually baseline corrected and exponentially line-broadened with a 1Hz window function using TOPSPIN 3.6.1 (Bruker) software. The spectra were then imported into Chenomx 8.4. A shim correction of 1.2 Hz was applied and the region from 4.56 ppm to 4.97 ppm was deleted to eliminate water resonance peaks. Peak assignment was performed using the Chenomx profiler tool fitting the spectral line to the proposed compounds in the standard Chenomx library. The efficacies of the extraction solvents, lysis methods and incubation temperatures were then compared using molecule concentration ratios and number of metabolites ratios between the two samples (Figure S3) (e.g.: I_E/M_ = I ethanol/I methanol).

The median, standard deviation (StDev) and coefficient of variance (CV) were all calculated to determine the reproducibility of the results. Any outliers were detected and removed from the analysis.

## 3. Results

In order to determine the optimal protocol to extract metabolites from *Blastocystis* ST7 for NMR analysis, a series of extraction solvents, lysis techniques and incubation temperatures were examined. The efficacy of each protocol was assessed using proton NMR and the peak intensity was compared using TOPSPIN 3.6.1 to determine which method extracted the highest concentrations of metabolites. We then developed an efficient, reproducible protocol to perform metabolomics studies on *Blastocystis* species and found that the extraction solvent and lysis method were the most important factors for metabolite extraction. The efficacy was optimised in four sets of experiments, which firstly compared solvents (MeOH versus EtOH), then compared methods (sonication vs. bead bashing) and finally the temperature regime used (−20 °C versus RT,) and (60 °C versus RT).

### 3.1. Comparison of Steps

Two analysis methods were used during the comparisons of pairs of processing steps to rank efficacy. These were molar concentration ratios C μM_A/B_ for processes A and B, as measured using the standard Chenomx metabolite library against the internal DSS standard. Secondly, the ratios of the raw number of detectable metabolites extracted N_A/B_ using the two processes (A and B), again using the Chenomx metabolite library. All analyses were made in pairs of samples in triplicates, and samples in the triplicate were denoted by Arabic numerals 1–3 and condition pairs by A and B. Therefore, for a comparison of methanol and ethanol, 1A–3A were ethanol samples and 1B–3B were methanol samples.

### 3.2. Extraction Solvent

The first part of this investigation focused on determining the most suitable extraction solvent (ethanol or methanol) for the extraction of Blastocystis from cultures.

**Figure 1:**
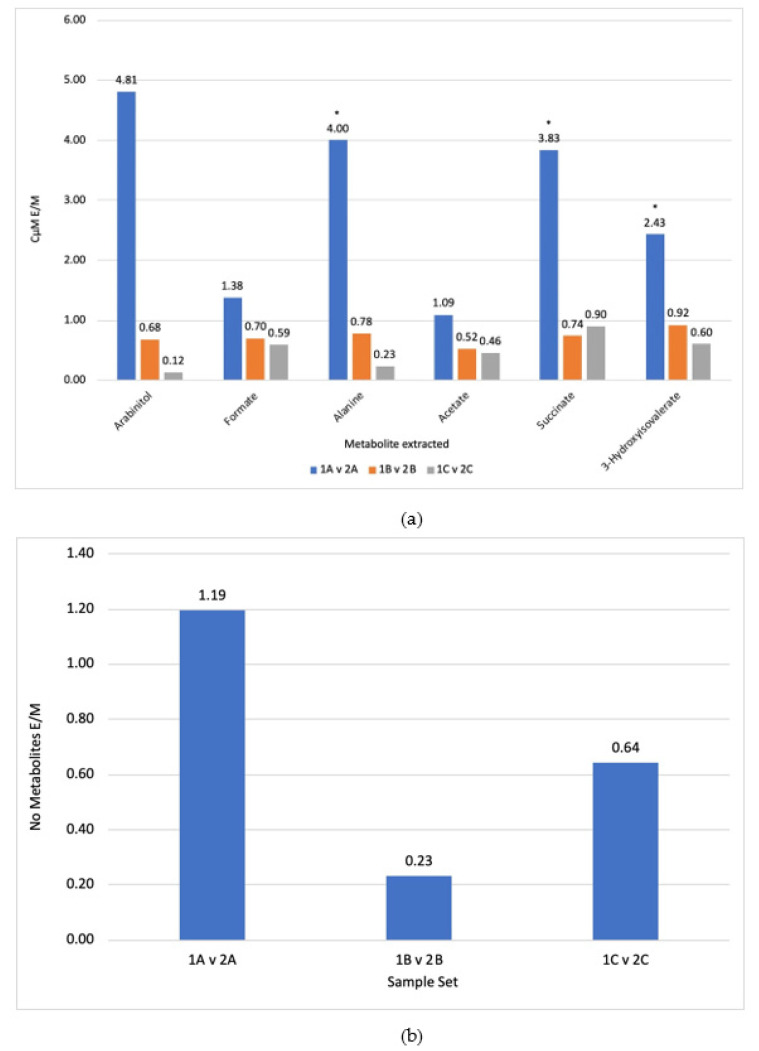
(**a**) Difference in metabolite concentrations between ethanol (1) and methanol (2) C μM _E/M_ extractions for the triplicates A–C. (**b**) Difference in the number of different metabolites extracted between ethanol (1) and methanol (2) extractions N_E/M_ for the triplicates. Numbers below 1.0 indicate an increased extraction in methanol, * = outliers, numbers above the bars indicate measured ratios.

Two sets of triplicates of metabolite extractions from Blastocystis cells were trialled using ethanol or methanol as an extraction co-solvent with water. The efficacies of the extraction solvents were compared using C µM_A/B_ and N_A/B_ between the two samples calculating the ratio of ethanol/methanol. The ethanol extractions were labelled sample 1A–1C and methanol extractions were labelled sample 2A–2C. The results of the extractions are shown in Figure 1a,b as C μM_E/M_ for a selected set of molecules and N_E/M_, respectively. The triplicates shown in Figure 1a show that extraction from ethanol and water vs. extraction from methanol and water produced two consistent results. Four molecules from the 1A vs. 2A sample set were identified as outliers (Figure 1a). The 1A vs. 2A sample set was also identified as an outlier for the number of molecules extracted. The reproducibility of the triplicates was measured by the CV (Table S1, Supplementary Information) and the CV improved as the outliers were removed (Figure S1, Supplementary Information). All the reproducible results were below one, with the exception of formate and acetate in the sample set 1A vs. 2A and sample set 1A vs. 2A for the number of molecules extracted. The CV for the number of molecules extracted was 0.7, showing poor reproducibility. These results suggest that methanol worked better than ethanol. All six of the selected metabolites produced values below one in two of the three sample sets, and two of the three sample sets produced values below one for the number of metabolites extracted. Taken together, the results suggest that methanol was the better extraction solvent.

### 3.3. Lysis Method

The lysis method for metabolite extraction was subsequently investigated as part of this experiment; here, samples which had been extracted with methanol (deemed the most suitable extraction solvent) were subjected to different lysis techniques.

**Figure 2:**
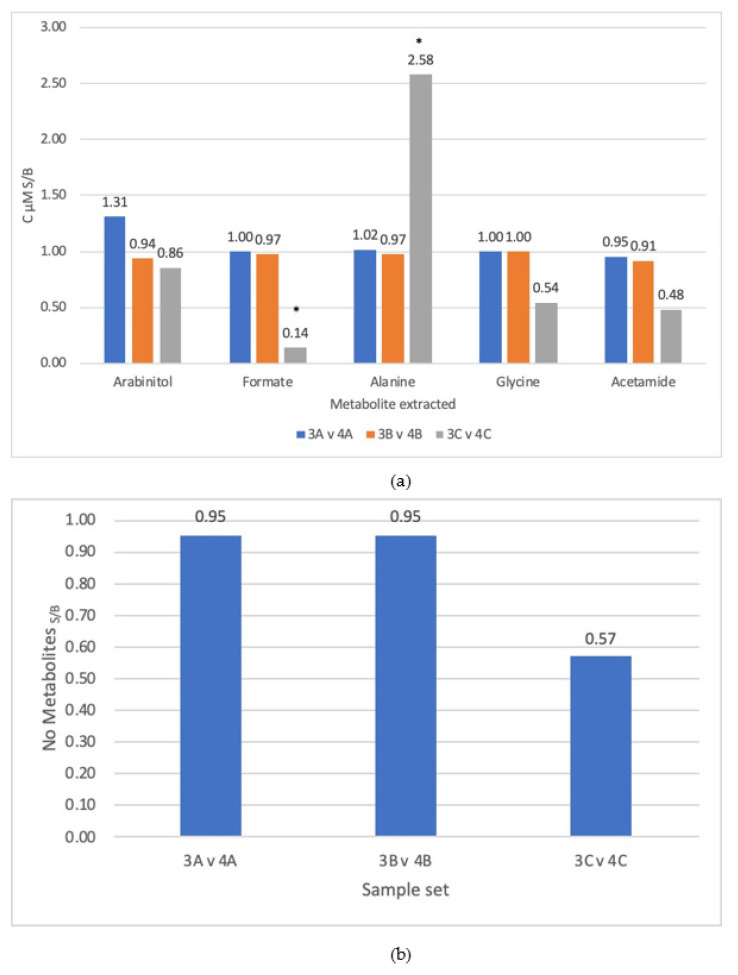
(**a**) Difference in concentrations between sonication (3) and bead bashing (4) C µMS/B lysis techniques for the triplicates A–C. (**b**) Difference in the number of different metabolites extracted between sonication (3) and bead bashing (4) lysis techniques NS/B for triplicates. Numbers below 1 indicate an increased extraction for bead bashing. * = outliers, numbers above the bars indicate measured ratios.

Two sets of triplicates of metabolite extractions from Blastocystis cells were examined with either bead bashing or sonication as the differing lysis methods. The efficacies of the lysis methods were compared using C µM_A/B_ and N_A/B_ between the two samples calculating the ratio of sonication/bead bashing. The sonicated extractions were labelled sample 3A–3C and bead-bashed extractions were labelled sample 4A–4C. The results of the extractions are shown in Figure 2a,b as ‘C μM _S/B_’ for a selected set of molecules and ‘N _S/B_’, respectively.

The triplicates show that for lysis by bead bashing vs. lysis by sonication, bead bashing produced more consistent results for the number of metabolites extracted, with all three triplicates being below one (Figure 2b). For the metabolite concentrations extracted, two metabolites were noted as outliers: alanine and formate for the pair sample set 3C vs. 4C (Figure 2a) and were removed and the CVs dropped from 0.7 to 0.02 and 0.6 to 0.03, respectively (Figure S2 and Table S2, Supplementary Information). All other peaks yielded three reproducible triplicates (Figure 2a). Of the reproducible triplicates, seven gave C μM_A/B_ ratios which were below 1.0 and five that were above; these produced no significant results on aggregate. The number of molecules extracted produced reproducible triplicates (Figure 2b) with no outliers and a CV of 0.27 (Figure 2b), all of which were below a ratio of 1.0. These results suggest that bead bashing was a more reliable method for lysis of Blastocystis when compared to sonication.

### 3.4. Incubation Temperature

Lastly, the final part of this investigation aimed at assessing the best incubation temperature for the extraction of metabolites from Blastocystis cultures. This part of the experiment used samples that had undergone extraction with methanol (extraction solvent) and bead bashing (lysis technique), chosen because they proved the most suitable methods, as described above.

**Figure 3:**
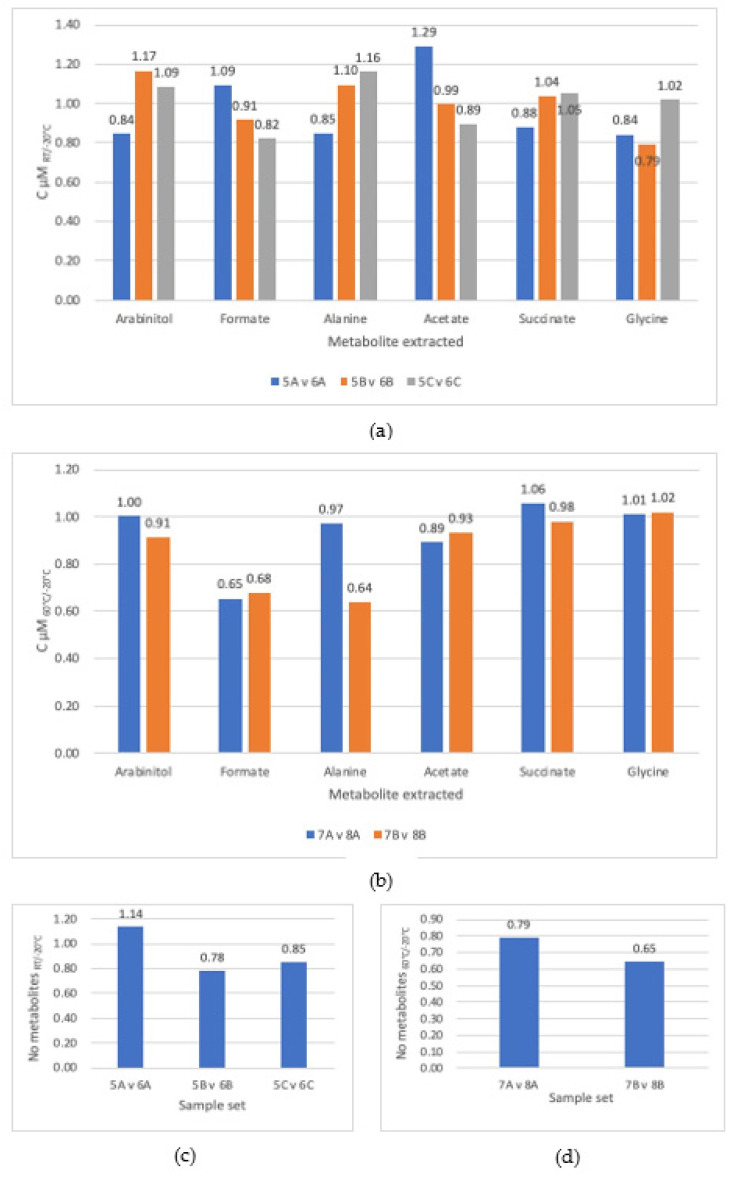
(**a**) Difference in concentrations between RT (5) and −20 °C (6) C µMRT/−20ºC incubation temperature for triplicates A–C. Numbers below 1 indicate an increased extraction for −20 °C incubation. (**b**) Difference in concentrations between 60 °C (7) and −20 °C (8) incubation temperatures for triplicates A–C. Number below 1 indicate an increased extraction for −20 °C incubation. (**c**) Difference in the number of different metabolites extracted between RT (5) and −20 °C (6) incubation temperatures. NRT/−20 °C for triplicates A–C. Numbers below 1 indicate an increased extraction for −20 °C incubation (**d**) Difference in the number of different metabolites extracted between 60 °C (7) and −20 °C (8) incubation temperatures N60 °C /−20 °C for triplicates A–C. Numbers below 1 indicate an increased extraction for −20 °C incubation. Numbers above the bars indicate measured ratios.

Two sets of triplicates of metabolite extractions from *Blastocystis* cells were trialled under the following incubation temperatures: −20 °C or room temperature (RT). The efficacies of the incubation temperatures were compared using C µM_A/B_ and N_A/B_ between the two samples calculating the ratio of RT/−20 °C. Results of the extractions are summarised in Figure 3a,c as C μM_RT_/−20 °C for a selected set of molecules and N_RT_/−20 °C, respectively. The triplicates produced show that incubation at −20 °C vs. incubation at RT produced consistent results, with no outliers (Table S3, Supplementary Information). All but one of the result medians were within 0.1 of 1 (Table S3, Supplementary Information), meaning no significant results were produced. The number of molecules extracted also produced consistent results with a CV of 0.21, but there were no significant differences between the two temperatures. Therefore, neither temperature appeared to be the more efficacious for metabolite extraction.

In addition to investigating the effect of RT and −20 °C incubation temperatures, a 60 °C incubation was also trialled. Two sets of duplicates of metabolites extracted from Blastocystis cells were included, using −20 °C or 60 °C as the incubation temperatures. The efficacies of the incubation temperatures were compared using C µM_A/B_ and N_A/B_ between the two samples calculating the ratio of 60 °C/−20 °C. The results of the extractions are shown in Figure 3b,d as C μM 60 °C/−20 °C for a selected set of metabolites and N _60/−20 °C_, respectively. Duplicates were executed for this test and produced consistent results. The CVs all ranged between 0.01 and 0.29, suggesting that all results were reproducible (Table S4, Supplementary Information). There were no significant differences between the different extraction temperatures. Additionally, the number of metabolites extracted produced reproducible results, with a CV of 0.14 (Figure 3d).

Overall, it was determined that temperature was not an important factor in metabolite extraction here. This means that performing the experiment at RT would be sufficient to extract metabolites from Blastocystis.

**Figure 4:**
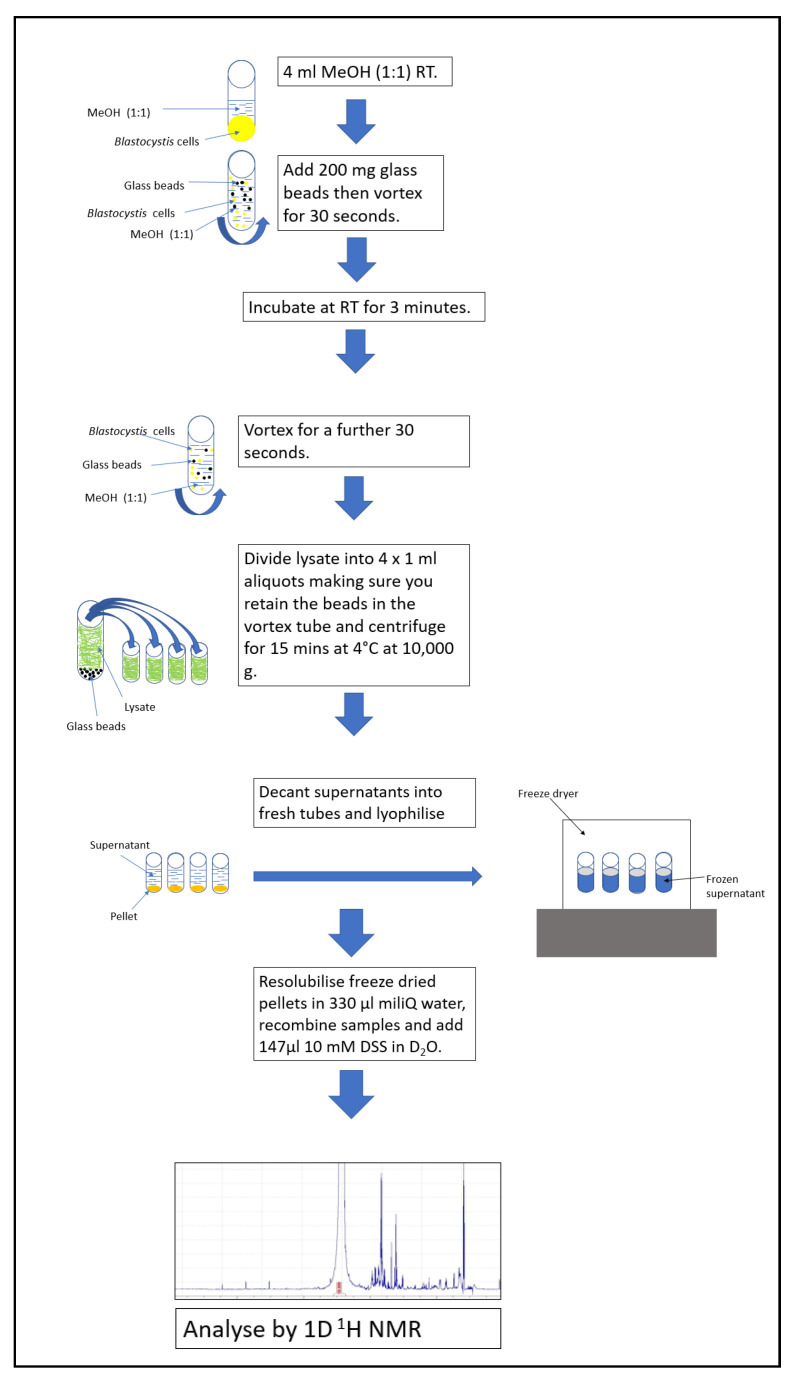
Final metabolite extraction protocol optimised by this study. Methanol is used as the extraction solvent, bead bashing as the lysis technique and incubation at RT.

The best extraction protocol (methanno/bead-bashing/RT) gave the 1D-^1^H-NMR spectrum shown in Figure 4, with Table S5, Supplementary Information containing the list of the most abundant molecules identified in this spectrum. Arabinitol and formate were the most abundant molecules. However, amino acids such as alanine and leucine were also be identified, along with molecules involved in Blastocystis energy metabolism such as acetate and succinate. Small sugars such as disaccharide trehalose and monosaccharide galactitol were identified, along with the lipid membrane component sn-Glycero-3-phosphocholine. Other molecules with biological roles such as betaine and malonate were also detected. Betaine has a role in regulating osmotic stress.

## 4. Discussion

Herein, we have described an efficient protocol to extract metabolites from *Blastocystis* ST7 in culture, thus allowing an overview of its metabolome by ^1^H-NMR analysis to be established for the first time. The findings can be summarized as follows: (1) methanol is a more effective extraction solvent when compared against ethanol; (2) bead bashing is a more effective lysis method than sonication; (3) incubation temperature is not a significant factor in metabolite extraction of *Blastocystis*; thus, performing the extraction at room temperature (RT) is sufficient. These data were collated to produce a series of steps to form an effective protocol to perform metabolite extraction on *Blastocystis* (Figure 4).

### 4.1. Methanol Was Determined to Be the Optimal Extraction Solvent

The results demonstrated that methanol was a more suitable solvent when compared against ethanol (Figure 1a). The molecule analysis produced six reproducible results: four of the molecules had one outlier and the other two had three reproducible results with one, which suggested ethanol was a better extraction solvent. All of the outliers and results that suggested ethanol was better came from a single sample (sample A). This could have been caused by an error in aliquot division when mixing a culture of cells, or homogeneity of the sample may not have been successfully achieved. The number of molecules extracted were consistent with metabolite concentration analysis, with sample set A being the only triplicate in which ethanol demonstrated better metabolite extraction than methanol. Overall, these results indicate that for *Blastocystis* ST7, methanol is a better extraction solvent. This is in contradiction to one past publication, in which a comparison between methanol and ethanol both produced similar results [29].

### 4.2. Bead Bashing Was Determined to Be the Optimal Lysis Method

Bead bashing was determined to be a more effective lysis technique when compared against sonication (Figure 2a). These are non-aggressive lysis techniques employed for *Blastocystis* as it does not possess a cell wall and is a single-celled organism, so cells are not connected by an extracellular matrix. One study by Geier et al. on *Caenorhabditis elegans* investigated different bead beating techniques, including some at cryogenic temperatures which produced successful results. A tissue homogenizer proved to be the most effective method here, yet it should be considered that *C. elegans* is a multicellular organism, meaning a more aggressive lysis technique is required [10]. Other research has demonstrated that cryopulveristation and tissue homogenisers were successful techniques for the lysis of mammalian cells [12,13]. However, sonication had proved successful in *Arabidopsis thaliana* [9], which has a cell wall and is tougher to break than *Blastocystis*. As sonication and bead bashing had both proved successful in tougher cells than *Blastocystis,* these two methods were selected. Bead bashing produced reproducible results (Figure 2a) against sonication, with only two selected peaks determined as outliers amongst all the samples. Nevertheless, the results of the extracted metabolite concentration ratios were not significant. The differences in concentrations of metabolite extracted ranged between 0.48 and 1.31 (Figure 2a) for most of the selected extracted metabolites, with the exception of formate and alanine in the 3C vs. 4C sample set, whose differences in concentration ranged between 0.14 and 2.58. The number of metabolites extracted produced three reproducible triplicates all suggesting that bead bashing was a better lysis technique than sonication and thus, bead bashing was consistently more successful than sonication.

### 4.3. Temperature Was Not an Important Factor in Metabolite Extraction

Incubation temperature was determined to not be a significant factor in successful metabolite extraction from *Blastocystis.* Additionally, as higher temperatures are more likely to facilitate chemical reactions, performing the experiment at room temperature may be essential for maintaining metabolite integrity. This is consistent with a past study by Beltran et al. [11]. However, it could also be the case that a 3-min incubation at the relevant temperature may not be long enough to have a sufficient effect and provides an avenue for future research into method optimisation. We would also like to emphasize that due to the nature and sensitivity of the organisms to oxygen, the objective was to minimise the extraction time to maintain sample integrity. RT against −20 °C (Figure 3a) produced a range of metabolite concentration ratios between 0.79 and 1.29. There were therefore no consistent, significant results and this was reproducible, suggesting that neither RT nor −20 °C was more successful. In past studies on human vein tissue and *C. elegans*, incubation at RT has been successfully performed [10,30], and similar experiments using *A. thaliana* demonstrated that successful extractions had been performed at −20 °C.

In the 60 °C incubation against the −20 °C incubation (Figure 3b), all of the extracted metabolite concentration ratios were between 0.64 and 1.06. All of the ratios were reproducible between the samples and there was no significant difference determined between them. For the number metabolites extracted ratios were both below 1.0, suggesting that −20 °C incubation was a more efficient incubation temperature to perform metabolite extraction than 60 °C. As RT was shown to be of similar efficacy to −20 °C, RT was selected as the best and most practical incubation temperature.

In summary, the most effective protocol determined by this study is shown in Figure 4. To summarize, this included methanol as the extraction solvent, accompanied by bead bashing and incubation at room temperature. Lyophilisation was used in each trial as a drying method and appeared to be a clean, consistent and successful drying technique. Although many of the results were reproducible, there were numerous outliers and, in some cases, only two reproducible results were produced amongst triplicates. For this reason, future work will aim to include more repeats in order to increase the reliability of the data. Therefore, for our final protocol quintuplets will be used, thus allowing the dismissal of one outlier, if necessary, to have successful triplicates.

The metabolites extracted by this protocol include amino acids such as alanine and leucine and molecules involved in energy metabolism such as acetate and succinate (Table S5, Supplementary Information). Additionally, a wide range of other molecules involved in biological processes such as betaine and malonate were present. The protocols trialled produced a range of metabolites numbering between 25 and 65. These were all polar molecules, as the solvents used target polar metabolites specifically. In the only other metabolomic study of a protozoan parasite, Vermathen et al. detected 31 different metabolites in *Giardia lamblia* using ^1^H HR-MAS NMR. However, they detected 22 amino acids (18 proteogenic and 4 non-proteogenic) which is at a higher abundance than what was detected here in *Blastocystis* [4]. However, molecules such as betaine and succinate which are involved in biological processes were not detected in *G. Lamblia* [4] but were detected in quite a high abundance in *Blastocystis*. This could be because of the two organisms’ different metabolisms, but also may be due to *Blastocystis* morphing into the cyst form and altering its metabolism subject to environmental changes.

Other NMR metabolomics studies of eukaryotic cells have demonstrated a similar number of metabolites to that extracted from *Blastocystis* at high concentrations. In a study on *Caenorhabditis Elegans* by Geier et al., 32 metabolites were detected at concentrations ranging between 2.48 mM and 5.73 mM [7]. Furthermore, in a study by Geier et al. on the avian liver, 52 polar metabolites were detected [10], and in a study on the rat liver by Lee et al., 30 metabolites were detected at concentrations ranging between 13.6 µM and 5.28 mM using methanol as an extraction solvent [8]. Bruno et al. extracted 38 metabolites from skeletal muscle using methanol and chloroform [9]. Methanol and chloroform form a two-layered solution with chloroform on top and methanol on the bottom. The polar metabolites migrate towards the methanol layer and the non-polar metabolites migrate towards the chloroform layer [9].

Even though we were unable to analyse a wider range of molecules, our established methodology was determined to be the most efficacious process from this study to use for the extraction steps for future metabolomics studies on *Blastocystis*. There are a wide range of metabolites which were not detected in this study which have been detected in past studies to map *Blastocystis*’ metabolism. Malate, oxaloacetate and succinyl-coA, for example, are all involved in *Blastocystis* energy metabolism and ATP generation, but were not detected using this extraction method [1]. Additionally, the production of amino acids isoleucine and serine have also been detected in past studies [1] but were not detected using this method. This could be down to *Blastocystis* morphing into cyst form and its metabolism becoming dormant but could also be down to the inefficiency of this method for extracting those specific metabolites.

## 5. Conclusions

In this study, we developed an efficient and robust protocol to extract and analyse polar metabolites from *Blastocystis*. We generated many ^1^H-NMR spectra to provide detail on the efficacy of each step of the protocol. This is the first extraction method described for NMR metabolomics analysis of *Blastocystis* species and it will spearhead future investigations to determine the metabolome of other *Blastocystis* subtypes, both in vitro, but also in vivo (e.g., stool metabolomic profiles). As such, this easy-to-use procedure could be applied to establish biomarkers in stool samples that could be subsequently used for (infectious) disease diagnosis.

## Figures and Tables

**Table 1:** Conditions of each experiment used to determine the best lysis method, incubation temperature and extraction solvent.

Experiment No.	Batch No.	Extraction Solvent	Lysis Method	Incubation Temp
1	1	4 mL EtOH (3:1) −20 °C	Sonication 3 × 30 s	3 min −20 °C
	2	4 mL MeOH (1:1) −20 °C		
2	1	4 mL MeOH (1:1) −20 °C	Bead Bashing–200 mg beads vortex 30 s	3 min −20 °C
	2	4 mL MeOH (1:1) −20 °C	Sonication 3 × 30 s	
3	1	4 mL MeOH (1:1) −20 °C	Sonication 3 × 30 s	3 min −20 °C
	2	4 mL MeOH (1:1) RT		3 min RT
4	1	4 mL MeOH (1:1) 60 °C	Sonication 3 × 30 s	3 min 60 °C
	2	4 mL MeOH (1:1) RT		3 min RT

## Supplementary Materials

The following are available online. Table S1: C μM_E/M_ for a selection of metabolites extracted from the ethanol vs. methanol experiment; Table S2: C μM_E/M_ for a selection of metabolites extracted from the sonication vs. bead bashing experiment; Table S3. C μM_E/M_ for a selection of metabolites extracted from the RT vs. −20 °C incubation experiment; Table S4. C μM_E/M_ for a selection of metabolites extracted from the 60 °C vs. −20 °C incubation experiment; Table S5. Metabolites extracted by the optimal extraction method and their concentrations. Figure S1: Reproducibility of the ethanol extractions against the methanol extractions-C.Vs of ethanol vs. methanol metabolites extracted with outliers and without outliers; Figure S2: Reproducibility of sonication lysis against bead-bashing lysis. C.Vs of sonication vs. bead bashing metabolites extracted with outliers and without outliers. Figure S3. Spectrum obtained from the optimal extraction protocol deduced from this study.

## Sample Availability

Not Available.
