# Supplementary material for: Establishing a Metabolite Extraction Method to Study the Metabolome of Blastocystis Using NMR
Source: Molecules. 2021 May 29;26(11):3285. doi: 10.3390/molecules26113285 (PMC8199492; doi:10.3390/molecules26113285)
Supplement: Supplementary file 1 [file molecules-26-03285-s001.zip › molecules-1225374-supplementary.pdf]

Table S1. C μM$_{E/M}$ for a selection of metabolites extracted from the ethanol vs methanol experiment. 1A, 1B and 1C = ethanol and 2A, 2B and 2C = methanol. The data is displayed as a metabolite concentration ratio using the 'equation n = C μM ethanol/C μM methanol' so numbers above 1 represent a positive result for Ethanol and numbers below 1 represent a positive result for methanol. The median, standard deviation = stdev and coefficient of variance = C.V were all calculated with and without outliers. Outliers in red.

	1A v 2A	1B v 2B	1C v 2C	Median	stddev	C.V	Median inc outliers	stddev inc outliers	C.V inc outliers
**Arabinitol**	4.81	0.68	0.12	0.40	0.39	0.98	1.87	2.56	1.37
**Formate**	1.38	0.70	0.59	0.89	0.43	0.48	0.89	0.43	0.48
**Alanine**	4.00	0.78	0.23	0.50	0.39	0.77	1.67	2.04	1.22
**Acetate**	1.09	0.52	0.46	0.69	0.35	0.51	0.69	0.35	0.51
**Succinate**	3.83	0.74	0.90	0.82	0.11	0.14	1.82	1.74	0.96
**3-Hydroxyisovalerate**	2.43	0.92	0.60	0.76	0.23	0.30	1.32	0.98	0.74

Table S2. C μM$_{E/M}$ for a selection of metabolites extracted from the sonication vs bead bashing experiment. 3A, 3B and 3C = sonication and 4A, 4B and 4C = bead bashing. The data is displayed as a metabolite concentration ratio using the 'equation n = C μM sonication/C μM bead bashing' so numbers above 1 represent a positive result for sonication and numbers below 1 represent a positive result for bead bashing. The median, standard deviation = stdev and coefficient of variance = C.V were all calculated with and without outliers. Outliers in red.

	3A v 4A	3B v 4B	3C v 4C	Median	stddev	C.V	Median inc outliers	stddev inc outliers	C.V inc outliers
**Arabinitol**	1.31	0.94	0.86	1.03	0.24	0.23	1.03	0.24	0.23
**Formate**	1.00	0.97	0.14	0.99	0.02	0.02	0.70	0.49	0.70
**Alanine**	1.02	0.97	2.58	1.00	0.03	0.03	1.52	0.91	0.60
**Glycine**	1.00	1.00	0.54	0.85	0.26	0.31	0.85	0.26	0.31
**Acetamide**	0.95	0.91	0.48	0.78	0.26	0.34	0.78	0.26	0.34

Table S3. C μM$_{E/M}$ for a selection of metabolites extracted from the RT vs -20°C incubation experiment. 5A, 5B and 5C = RT and 6A, 6B and 6C = -20°C. The data is displayed as a metabolite concentration ratio using the 'equation n = C μM RT/C μM -20°C so numbers above 1 represent a positive result for RT incubation and numbers below 1 represent a positive result for-20°C incubation. The median, standard deviation = stdev and coefficient of variance = C.V were all calculated.

	5A v 6A	5B v 6B	5C v 6C	Median	Stddev	C.V
**Arabinitol**	0.84	1.17	1.09	1.03	0.17	0.16
**Formate**	1.09	0.91	0.82	0.94	0.14	0.15
**Alanine**	0.85	1.10	1.16	1.03	0.17	0.16
**Acetate**	1.29	0.99	0.89	1.06	0.21	0.19
**Succinate**	0.88	1.04	1.05	0.99	0.10	0.10
**Glycine**	0.84	0.79	1.02	0.88	0.12	0.13

Table S4. C μM$_{E/M}$ for a selection of metabolites extracted from the 60°C vs -20°C incubation experiment. 7A, 7B and 7C = 60°C and 8A, 8B and 8C = -20°C. The data is displayed as a metabolite concentration ratio using the 'equation n = C μM 60°C/C μM -20°C so numbers above 1 represent a positive result for 60°C incubation and numbers below 1 represent a positive result for-20°C incubation. The median, standard deviation = stdev and coefficient of variance = C.V were all calculated.

	7A v 8A	7B v 8B	Median	stddev	C.V
**Arabinitol**	1.00	0.91	0.96	0.06	0.07
**Formate**	0.65	0.68	0.67	0.02	0.03
**Alanine**	0.97	0.64	0.81	0.24	0.29
**Acetate**	0.89	0.93	0.91	0.03	0.03
**Succinate**	1.06	0.98	1.02	0.05	0.05
**Glycine**	1.01	1.02	1.01	0.01	0.01

Table S5. Metabolites extracted by the optimal extraction method and their concentrations.

Metabolite no	Metabolite	Concentration (μM)	Chemical shifts (ppm)
1	Arabinitol	15.9	3.56, 3.66, 3.66, 3.68, 3.75, 3.84, 3.93
2	Formate	11.1	8.44.
3	Alanine	6.9	1.47, 3.78
4	Trehalose	6.8	5.18, 3.85, 3.8, 3.76, 3.44
5	Acetate	5.5	1.91.
6	sn-Glycero-3-phosphocholine	5.1	3.21, 3.61, 3.66, 3.87, 3.92, 3.94, 4.32
7	Betaine	5	3.26, 3.89
8	Cadaverine	4.8	1.46, 1.71, 3.02
9	Ethylene glycol	3.2	3.66.
10	Galactitol	2.9	3.68, 3.97
11	Galactonate	2.7	3.64, 3.69, 3.71, 3.97, 4.27
12	Leucine	2.1	0.95, 0.96, 1.68, 1.7, 1.73, 3.73
13	1,3-Diaminopropane	2.1	2.07, 2.08, 3.11, 3.13,
14	Malonate	1.4	3.12.
15	Succinate	1.2	2.4.

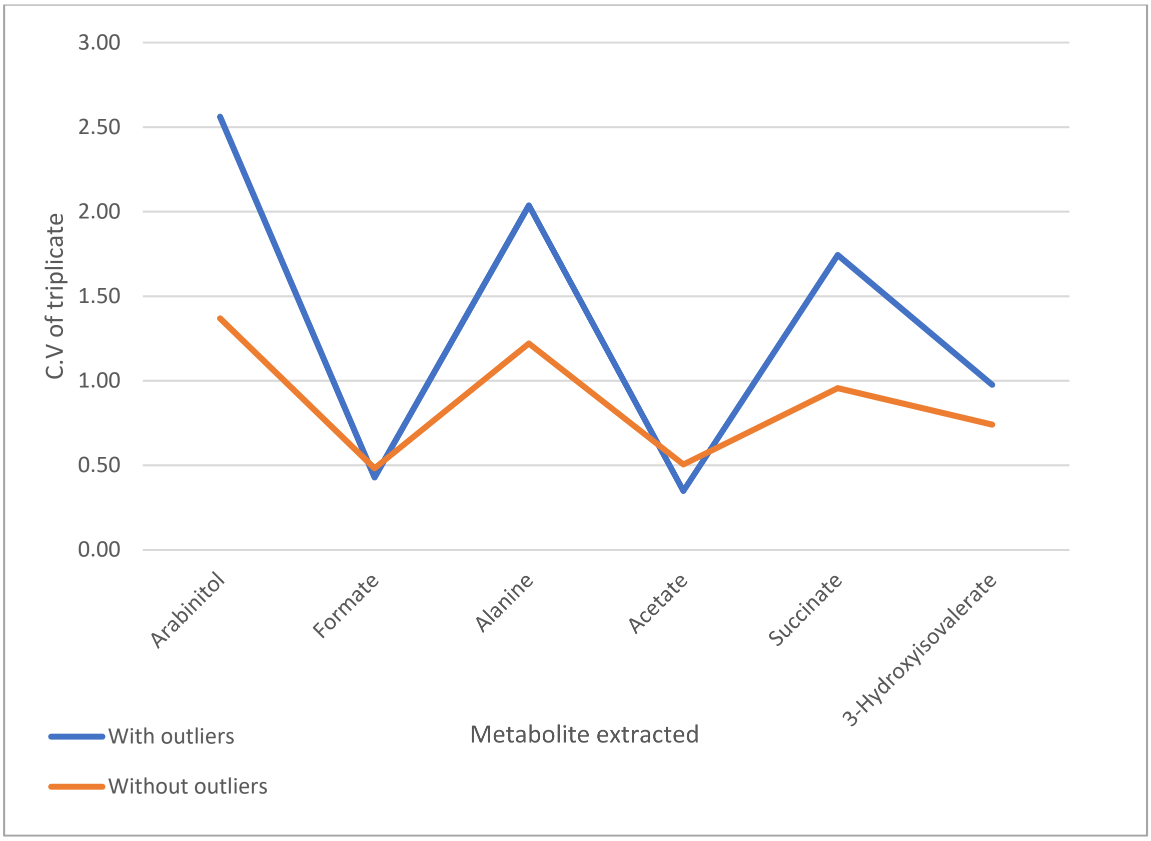

Figure S1. Reproducibility of the ethanol extractions against the methanol extractions - C.Vs of ethanol vs methanol metabolites extracted with outliers and without outliers.

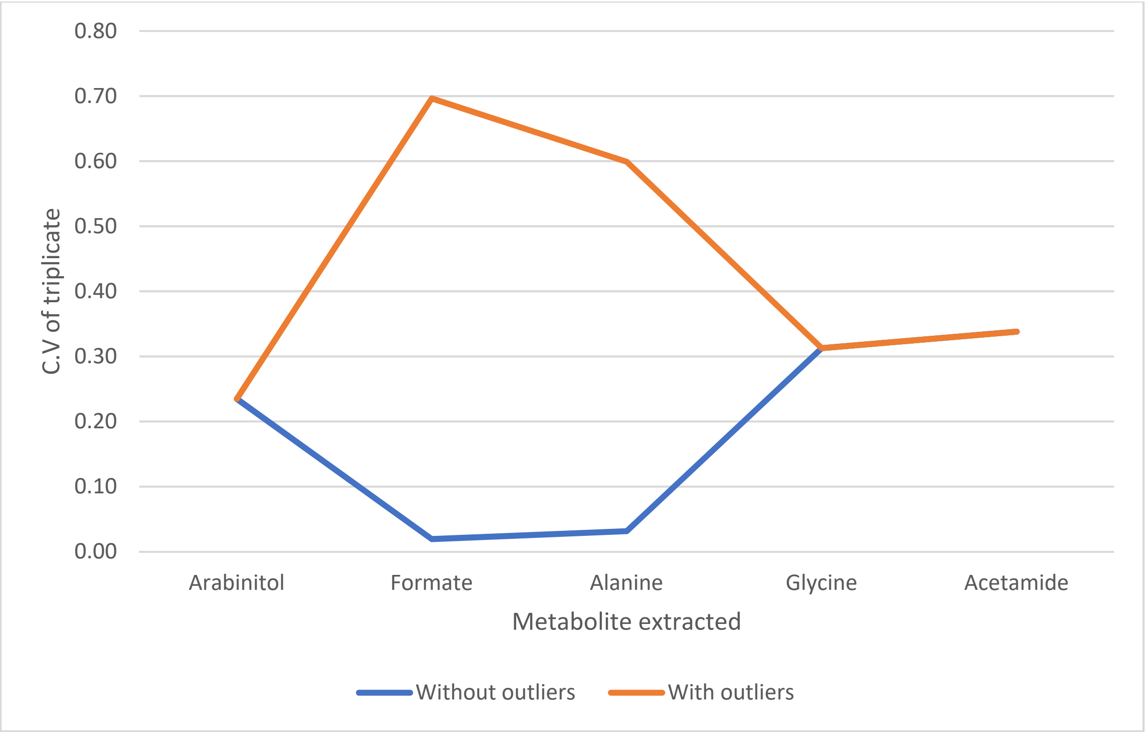

Figure S2. Reproducibility of sonication lysis against bead-bashing lysis. C.Vs of sonication vs bead bashing metabolites extracted with outliers and without outliers.

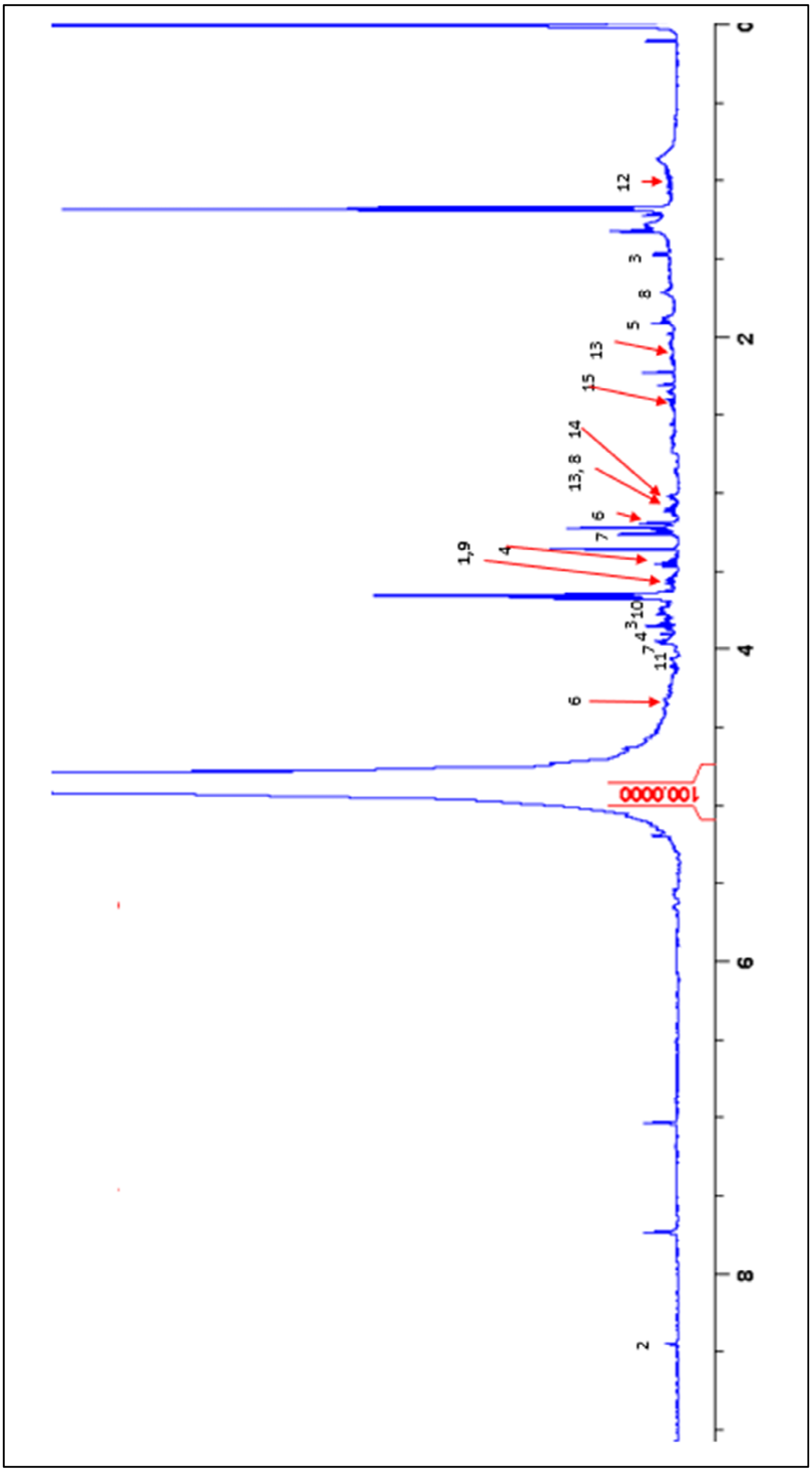

Figure S3. Spectrum obtained from the optimal extraction protocol deduced from this study. Using methanol as the extraction solvent, bead bashing as the lysis method and incubation at RT.
